# Astronomical Units to Merge as UK Cows Face Bloody Purge

**DOI:** 10.1100/tsw.2001.45

**Published:** 2001-04-20

## Abstract

Nature leads this week with a story about a proposal to fuse space-based astronomy funded by NASA with ground-based astronomy funded by the National Space Foundation (NSF). Science starts the week with a successs story about attempts to slow the spread of foot-and-mouth disease.

When it comes to paying for it, does it matter if your telescope is on the ground or orbiting in space? Astronomers say yes and the Bush administration says no, reports *Nature* in their lead story this week. (*Science* did not cover this story.) Virtually all funding for U.S. astronomy comes from either NASA or the NSF. The NSF spends most of its annual budget of $156 million on ground-based astronomy while NASA shoots around $1 billion a year into space on board projects like the Hubble Space Telescope and the Chandra X-ray Observatory, reports *Nature*. But the cost of ground-based telescopes is skyrocketing and the Bush administration believes that the two programs should be combined to take advantage of NASA's experience managing high-cost missions. Astronomers are not so sanguine. At a meeting of the National Academy of Science's Committee on Astronomy and Astrophysics last week in Washington, D.C., prominent scientists argued that the mission-oriented style NASA uses to get high-tech optical hardware into space would be incompatible with NSF's commitment to the whims of basic science. Despite the fears, reports *Nature*, astronomers long accustomed to tight NSF budgets might be seduced by NASA's financial largesse.

Foot-and-mouth disease is still spreading through European livestock, but there are indications that a more aggressive slaughter policy implemented in the U.K. has turned the tide in the fight against the disease, reports *Science* in their lead story for this week. (*Nature* did not cover this story.) Instead of allowing three days to elapse between diagnosing the disease in a herd and slaughtering the animals, government officials followed the advice of three computer models and reduced the time lag to 24 hours. They also began implementing a procedure called "ring-culling" in which all animals in farms adjacent to an infected herd are killed. The bloody response appears to be slowing the spread of foot-and-mouth, reports *Science*, but it has fanned the flames of a different debate. Prior to 1991, the UK vaccinated for foot-and-mouth but they dropped the policy to encourage exports to the U.S. and Japan. Both countries refuse to accept vaccinated animals for fear that they are "silent carriers" of the disease. Now that grisly flesh-and-bone funeral pyres are sprouting up all over the country — more than 1.1 million animals have been killed in the current epidemic — officials are reconsidering their fear of needles, reports *Science*. The E.U. is already permitting "emergency vaccination" of animals in the most affected area in the Netherlands, which faces its own small outbreak of 25 cases, reports *Science*.
